# A Comparison between Silver Nanosquare Arrays and Silver Thin-Films as a Blood Cancer Prognosis Monitoring Electrode Design Using Optical and Electrochemical Characterization

**DOI:** 10.3390/nano11113108

**Published:** 2021-11-18

**Authors:** Nasori Nasori, Ulya Farahdina, Vinda Zakiyatuz Zulfa, Miftakhul Firdhaus, Ihwanul Aziz, Darsono Darsono, Dawei Cao, Zhijie Wang, Endarko Endarko, Agus Rubiyanto

**Affiliations:** 1 Laboratory Medical Physics and Biophysics, Department of Physics, Faculty of Sciences and Data Analytic, Institut Teknologi Sepuluh Nopember, Surabaya 60111, Indonesia; ulyafarahdina06@gmail.com (U.F.); vzakiyatuz@gmail.com (V.Z.Z.); mfirdauz8@gmail.com (M.F.); endarko@physics.its.ac.id (E.E.); arubi@physics.its.ac.id (A.R.); 2 Occupational and Safety Department, Nahdlatul Ulama University of Surabaya, Surabaya 60237, Indonesia; 3 Center for Accelerator Sciences and Technology, Yogykarta 60101, Indonesia; ihwanul@batan.go.id (I.A.); b_darsono@batan.go.id (D.D.); 4 Department of Physics, Faculty of Sciences, University of Jiangsu, Zhenjiang 212013, China; dwcao@ujs.edu.cn; 5 Semiconductor Materials Science Key Laboratory, Semiconductors Institute, Chinese Sciences Academy, Beijing 100083, China; wangzj@semi.ac.cn

**Keywords:** blood cancer, biomedical, electrode, monitoring, nanosquare, silver, thin films

## Abstract

The development of silver (Ag) thin films and the fabrication of Ag nanosquare arrays with the use of an anodic aluminum oxide (AAO) template and leaf extracts were successfully carried out using the DC sputtering and spin coating deposition methods. Ag thin films and Ag nanosquare arrays are developed to monitor cancer prognosis due to the correlation between serum albumin levels and prognostic factors, as well as the binding of serum albumin to the surface of these electrodes. Nanosquare structures were fabricated using AAO templates with varying diameters and a gap distance between adjacent unit cells of 100 nm. The nanosquare array with a diameter of 250 nm and irradiated with electromagnetic waves with a wavelength of around 800 nm possessed the greatest electric field distribution compared to the other variations of diameters and wavelengths. The results of the absorption measurement and simulation showed a greater shift in absorption peak wavelength when carried out using the Ag nanosquare array. The absorption peak wavelengths of the Ag nanosquare array in normal blood and blood with cancer lymphocytes were 700–774 nm and 800–850 nm, respectively. The electrochemical test showed that the sensitivity values of the Ag thin-film electrode deposited using DC sputtering, the Ag thin-film electrode deposited using spin coating, and the Ag nanosquare array in detecting PBS+BSA concentration in the cyclic voltammetry (CV) experiment were 1.308 µA mM^−1^cm^−2^, 0.022 µA mM^−1^cm^−2^, and 39.917 µA mM^−1^cm^−2^, respectively. Meanwhile, the sensitivity values of the Ag thin film and the Ag nanosquare array in detecting the PBS+BSA concentration in the electrochemical impedance spectroscopy (EIS) measurement were 6593.76 Ohm·cm^2^/mM and 69,000 Ohm·cm^2^/mM, respectively. Thus, our analysis of the optical and electrochemical characteristics of Ag thin films and Ag nanosquare arrays showed that both can be used as an alternative biomedical technology to monitor the prognosis of blood cancer based on the concentration of serum albumin in blood.

## 1. Introduction

The early detection of tumours is vital for the treatment of cancer and can significantly increase the chance of survival by up to 12.4% [1,2]. To date, there have been numerous methods developed for the detection of blood cancer [3,4,5]. Prabhakar et al. [6] used the FTIR method to detect blood cancer and showed that blood with cancer had a greater absorption peak of 0.01 a.u than that of normal blood. Nevertheless, the absorption peaks of biomolecules may overlap with one another [7]. Studies using the electrochemical technique have been carried out to detect various cancer biomarkers [8]. This type of experiment requires specific protein biomarkers for each type of cancer [9,10]. Research conducted by Clement et al. showed that the level of serum albumin in patients is an independent prognostic factor in the diagnosis of leukaemia [11]. Leukaemia patients with low levels of serum albumin have a lower overall survival rate compared to patients with normal levels of serum albumin [12]. The reactive protein contained in commercial human serum albumin (HSA) is said to be very similar to the protein in bovine serum albumin (BSA) due to both HSA and BSA having the same structure and molecular weight [13,14].

Biosensor electrodes have been widely developed to detect various abnormalities in blood. The shapes and sizes of electrode constituents are varied in an attempt to obtain electrode biosensors that have good performance, such as the manufacturing of nanodots, nanotubes, etc. [15,16,17]. A review on biosensors conducted by Parthasarathy et al. stated that nanostructures are better electrochemical biosensors compared to thin films because the biocompatibility of nanostructures is higher than that of thin films [18]. A nanostructure is a form of electrode that has the potential to become a biosensor due to its high sensitivity and accuracy in detecting cancer biomarkers and its nature attractiveness with molecules [19]. Gold (Au) is commonly used as a biosensor [20,21,22]. However, Ag is also a good candidate for biosensors. The study carried out by Lismont and Dreesen [23] showed that Ag was better than Au when used as a surface plasmon resonance (SPR)-based biosensor. Ag can be used as an alternative basic material of biosensors due to its high conductivity, its ability to sense weak bioelectrical signals, the fact that it is not polarized with the occurrence of an electric current, and highly photocatalytic nature [24,25].

To date, the development of electrodes to monitor the prognosis of blood cancer patients is still rare [12,26,27]. The use of Ag nanostructures with AAO templates is a simple and inexpensive method to obtain a blood cancer prognosis monitoring sensor [28,29]. This study found that Ag nanosquare arrays and Ag thin films with different biological material media possess different optical and electrochemical characteristics. This study also investigates the impact of the gap distance between adjacent unit cells in Ag nanosquare arrays on the optical characteristics of the electrode system by means of an electric field distribution simulation with an initial amplitude of 1 V/m. Therefore, the actualization of an Ag nanosquare array electrode and an Ag thin film electrode is proof that both Ag nanosquare arrays and Ag thin films can be used to monitor the prognosis of blood cancer based on the concentration of serum albumin using spectroscopic and electrochemical methods.

## 2. Materials and Methods

### 2.1. Materials and Chemicals

Acetone (C_3_H_6_O) (99.8%), methanol (CH_3_OH) (99.8%), sodium hydroxide (NaOH) (99.8%), phosphoric acid (H_3_PO_4_) (70%), and distilled water (DI-H_2_O) were obtained from SIP, and the hypericin leaf extract was obtained from Sigma Aldrich. (Singapore, Singapore) Ag (99.99%), was obtained from Goodfellow and the Ag enhancer solution A was bought from Sigma Aldrich. Thick Al foil (99.99%) was acquired from Advance Tapes (Surabaya, Indonesia), while indium tin oxide (ITO) glass with a transmittivity of 86%, a resistance of 30–40 Ω, and a thickness of 1.1 mm was bought from Ali Laboratory and Mechanics (Surabaya, Indonesia). Phosphate buffer saline (PBS) and bovine serum albumin (BSA) were purchased from MaxLab (Tangerang, Indonesia) and Sigma Aldrich (Darmstadt, Germany).

### 2.2. Fabrication of AAO

The fabrication of aluminium pore structures had the same procedure as described in [30]. At the imprinting phase, 10 kP of pressure was applied to the aluminium sheets for 3 min. The anodization procedure was carried out at a temperature of 4 °C for 1 h with a voltage of 160 V. The result of this process is known as the AAO template. The AAO template was then submerged in a 5 wt% H_3_PO_4_ solution in order to expand the AAO pores.

### 2.3. Ag Deposition

Ag deposition on ITO glass was carried out using 2 methods, namely spin coating and DC sputtering. Ag deposition was carried out at BATAN Yogyakarta, Indonesia. For the DC sputtering process, the Ag sputtering target was placed at a distance of 25 mm from the substrate. Subsequently, the base pressure of the DC sputtering tube was set to 10^−2^ Pa. Pure argon gas (99.99%) was inserted into the sputter chamber through the valve with a constant pressure of 60 Pa. The substrate retainer functioned as an anode while the Ag slab functioned as a cathode. The voltage applied between the two electrodes was 2 kV. The durations of the Ag depositions using DC sputtering method were 10 s and 15 s. The spin coating process was carried out for 30 s with a rotation speed of 6000 rpm, while the annealing process was carried out at a temperature of 60 °C. Spin coating of the Ag thin film was carried out using 0.15 mL of Ag in a Hypericin extract solution with a volume ratio of 1:3.

### 2.4. Ag Characterization

The analysis to find out the phase and composition of the Ag nanostructure was carried out using X’pert Pro X-ray Diffraction (XRD). The morphology and microstructure analysis were carried out using the Phenom scanning electron microscope equipped with Thermo Scientific ProX-G6 (Institut Teknologi Sepuluh Nopember, Surabaya, Indonesia) energy-dispersive X-ray spectroscopy (EDS). The medium used to analyse nanostructure activities was PBS and a mixture of PBS-BSA. The PBS-BSA solution was made by dissolving PBS and BSA in 200 mL of distilled water. The concentrations of the solutions were varied at 0.12 mM, 0.14 mM, 0.16 mM, 0.18 mM, and 0.2 mM. Ag nanosquare arrays and Ag thin films were placed in both media in which their optical characteristics were analysed using a Thermo Scientific GENESYS 10S UVVis Spectrophotometer (Waltham, MA, USA). The performance measurement of the electrochemical medium was carried out using a CorrTest CS2350 potensiostat (Wuhan, China). The CV and EIS measurements used platina sheets as a counter electrode, Ag/AgCl as a reference electrode, and a working electrode for the Ag thin films and Ag nanosquare arrays on the ITO glass. The working electrode was activated with 60 cycles (CV) at a scanning speed of 100 mV/s in a NaOH 0.1 M solution. Next, the Ag nanosquare array electrode was used with the PBS and BSA medium with a scan rate between 50 and 150 mV/s.

### 2.5. Simulation Session

The finite difference time domain (FDTD) electric field distribution simulation was carried out using the Comsol Multiphysics^®^ version 5.4 in Laboratory Medical Physics and Biophysics, Department of Physics, Faculty of Sciences and Data Analytic, Institut Teknologi Sepuluh Nopember, Surabaya 60111, Indonesia [25]. The inputs to the electric field distribution simulation on the Ag thin films and Ag nanosquare arrays were the electromagnetic wavelength and the index of refraction of each material as a function of wavelength. The components that comprised the model were Ag, glass, and the medium. The media used in this simulation were blood and T lymphocyte as a blood cancer biomarker with the index of refraction as a function of wavelength [9,31,32]. Electromagnetic waves were shot through the top side of the medium in which the wavelength was varied between 300 and 1000 nm. The electric field distribution simulation was carried out using thin films and nanostructure models with diameters varying between 100 and 300 nm. The results of the simulation using Comsol Multiphysics^®^ version 5.4 are 3D figures of the electric field distribution of Ag thin films and Ag nanosquare arrays, the transmittance values, the absorbance values, and the total electric and magnetic energies as a function of wavelength.

## 3. Results and Discussion

### 3.1. Structure Analysis

**Figure 1:**
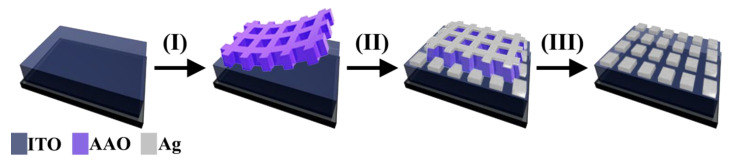
The fabrication scheme of the Ag nanosquare array. Step I: manually transfer UTAM on top of ITO. Step II: deposit Ag on UTAM using the DC sputtering method. Step III: remove UTAM using clear tape.

The fabrication of AAO using the imprinted method is a simple way to produce a well-ordered arrangement with structure control and a simple construction. This study fabricates Ag nanosquare array electrodes using AAO templates by means of the imprinted method. Figure 1 shows a schematic diagram of the steps in the fabrication of an Ag nanosquare array. The first step is the sterilization of ITO glass used as a substrate. This was achieved by placing an ultra-thin alumina mask (UTAM) substrate that had already been cleaned. UTAM is a template made by using the AAO process with Al used as the basic material. The UTAM was placed on top of the conductive layer of the ITO glass. While still submerged in the solution, ITO glass with UTAM placed on top was dried with water and dry tissue. The second step was the deposition of Ag on ITO/UTAM using the DC sputtering method and argon gas as the sputter. Ag atoms enter the pores of UTAM and are arranged to become an Ag nanosquare array. Finally, the templates of the ITO/UTAM specimens that were deposited by Ag were removed using clear tape, thus leaving an arrangement of an Ag nanosquare array, as shown in step III of Figure 1.

**Figure 2:**
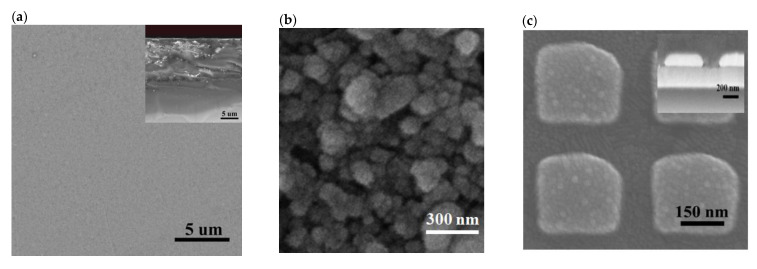

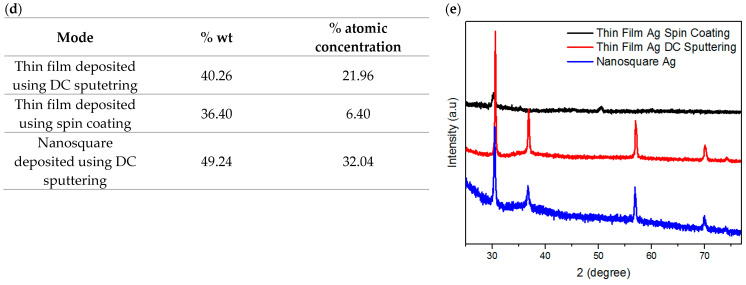
SEM images of (**a**) top view and side view of the Ag thin-film deposited using DC sputtering, (**b**) top view of the Ag thin-film deposited using spin coating, (**c**) top view and side view of the arranged Ag nanosquares array, (**d**) the EDS analysis results the Ag nanosquare array and Ag thin-films deposited using DC sputtering and spin coating, and also (**e**) XRD measurement result of the Ag thin-film and Ag nanosquare array electrodes [36].

A comparison between Ag thin films deposited using DC sputtering and spin coating and the Ag nanosquare array was conducted to determine the effectiveness and performance of these electrodes. SEM images of Ag thin films and the Ag nanosquare array are shown in Figure 2a–c. The thin films deposited using the DC sputtering method possessed a more homogeneous and even structure compared to the thin films deposited using the spin coating method that used leaf extracts as shown in Figure 2a,b, respectively. This is due to the Ag agglomeration in the extraction results of the spin coating method that used leaves, which caused the structure of the thin films to be less homogeneous. The arrangement of the Ag nanosquare array is relatively well-ordered due to the high structure control of the nanoimprinted AAO template. The Ag nanosquare array has a diameter of around 250 nm and an almost equivalent gap distance between adjacent unit cells of around 100 nm. The dimensions of the Ag nanosquare array were chosen according to the results of the preliminary simulation in which the Ag nanosquare array with a diameter of 250 nm showed a difference in peak wavelength and maximum absorbance intensity, as shown in Figure S1. The advantage of this nanoimprinted AAO pattern is that the nanostructure enhances the electrochemical signal of biocatalytic events occurring at the electrode/electrolyte interface for the purpose of biosensing [33].

The EDS analysis was carried out to determine the layer deposited on the substrate. Figure 2d shows the percentage of Ag atoms deposited on the nanosquare array and also the percentage of Ag atoms deposited using DC sputtering and spin coating on thin films. The main components of thin films based on the EDS results were Si, In, O, and Ag. The substrate used for the electrodes was ITO glass. Another component identified in the EDS analysis results was Ag. The atomic concentration and molecular weight of Ag in the thin film deposited using DC sputtering were 21.96% and 40.26%, respectively, which are higher than those of the other components. The high atomic concentration and molecular weight of this thin film indicated that Ag had been deposited on the ITO glass substrate. The concentration of Ag molecules was higher in the Ag thin film deposited using DC sputtering than the concentration of Ag molecules in the Ag thin film deposited using spin coating. This is influenced by the remaining leaf extracts that were bounded to the thin film and was proven by the presence of F and C atoms in the EDS analysis. The gaps between unit cells of the nanosquare array also caused the percentage of Ag atoms in the Ag thin film deposited using DC sputtering to be higher than that in the nanosquare array.

Figure 2e shows the results of the XRD data analysis of thin films deposited using DC sputtering and spin coating. The presence of sharp peaks in the diffraction pattern shows that the atom components of the electrode formed a crystalline phase. The qualitative analysis that was carried out shows that two phases were formed, namely ITO as a substrate and Ag as a layer deposited on top of the substrate. Based on the experiment carried out by Parsianpour [34], peaks in the diffraction pattern of ITO glass possessed Miller index values of (222), (400), (440), and (622). The crystal structure that was formed on the ITO glass was a simple cubic structure with a side length of 10.12 Å. The size of the ITO crystal on the thin film was relatively large, proven by the occurrence of a high peak in the diffraction pattern of the structure. Peaks in the diffraction pattern of Ag indicated that Ag deposited on ITO glass had Miller index values of (111) and (220). The peaks in the XRD pattern of Ag deposited using spin coating were relatively lower and wider than those deposited by DC sputtering. This proves that the size of the Ag crystal on the thin film deposited by spin coating was relatively smaller. Low crystallinity may indicate a less effective charge transport in thin films deposited using spin coating [35].

### 3.2. Electric Field Distribution and Optical Character Analysis

The electric field distribution was simulated on thin films and nanosquare arrays that had diameters of 300, 250, 200, 150, and 100 nm. Ag thin films and nanosquare arrays were placed on the glass substrate. The top part of Ag thin films and nanosquare arrays were coated with blood and blood with cancer. The electric field distribution simulation was carried out using the FDTD method and electromagnetic waves with a wavelength between 300 and 1000 nm. The electric field distribution simulation with the presence of electromagnetic waves was carried out based on the Gauss Law and Ampere Law equation; thus, the electric field of each component of the electrode was very dependent on their permittivity and permeability values [37].

**Figure 3:**
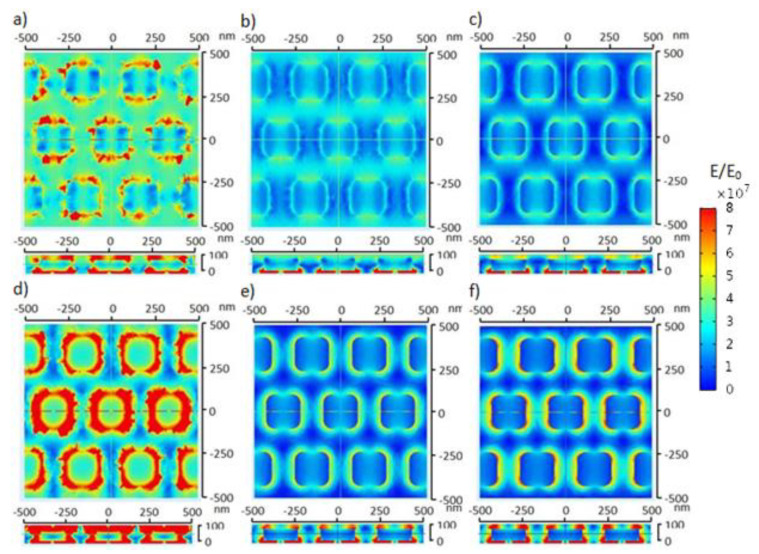
The top view and side view of the electric field distribution from the simulation results of Ag nanosquare arrays with a diameter of 250 nm irradiated with electromagnetic waves with wavelengths of (**a**) 500 nm, (**b**) 600 nm, (**c**) 700 nm, (**d**) 800 nm, (**e**) 900 nm, and (**f**) 1000 nm.

The top view and side view of the electric field distribution of Ag nanosquare arrays are shown in Figure 3. Electromagnetic waves were perpendicularly shot on the top part of the medium, causing a high electric field value for the top part of the medium. An electric field distribution pattern was formed due to the presence of electromagnetic waves. The electromagnetic waves given to the electrode were gradually distributed through the medium, Ag thin film or nanosquare array, and the glass substrate. The electric field distribution of each electrode had different values due to the difference in the natures of the constituent components of the electrode in passing on and reflecting the electromagnetic waves and also absorbing energy from the electromagnetic waves. A bigger electric field on the medium is caused by a much smaller relative permittivity value of the medium compared to the other components of the electrode [38]. Based on the simulation of the electric field distribution, the nanosquare array with a diameter of 250 nm possessed a much larger electric field distribution compared to the other nanosquare arrays.

**Figure 4:**
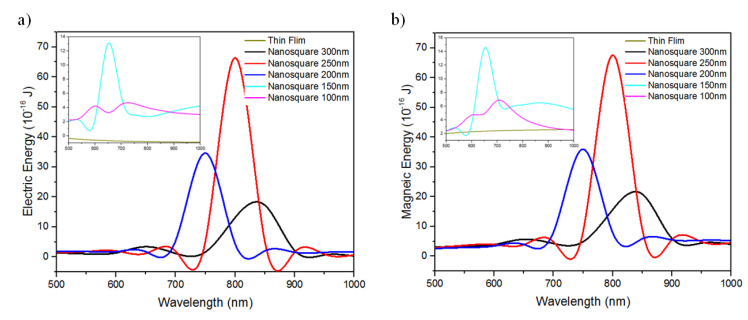
Simulation result of (**a**) electric energy and (**b**) magnetic energy from Ag thin films and Ag nanosquares with diameters of 100, 150, 200, 250, and 300 nm.

The electric field distribution can also be identified from the total electric and magnetic energy on Ag thin films and nanosquare arrays. Figure 4 shows the total electric and magnetic energy on Ag thin films and nanosquare arrays irradiated with electromagnetic waves with wavelengths between 500 and 1000 nm. The maximum total electric energy value was 6.64 × 10^−15^ Joule while the maximum total magnetic energy value was 6.76 × 10^−15^ Joule. Both maximum values of the total electric and magnetic energy values were obtained from the nanosquare that had a diameter of 250 nm and was irradiated with electromagnetic waves with a wavelength of 800 nm. The next highest total electric and magnetic energy values obtained were 3.46 × 10^−15^ Joule dan 3.59 × 10^−15^ Joule, respectively. These values were obtained from the nanosquare array that had a diameter of 200 nm and was irradiated with electromagnetic waves with a wavelength of 750 nm. The parameters, namely the wavelength and diameter of the nanosquare array, that produced the maximum electric field intensity can be used to improve the performance of the electrode because it directs the molecules towards the electrode [39].

**Figure 5:**
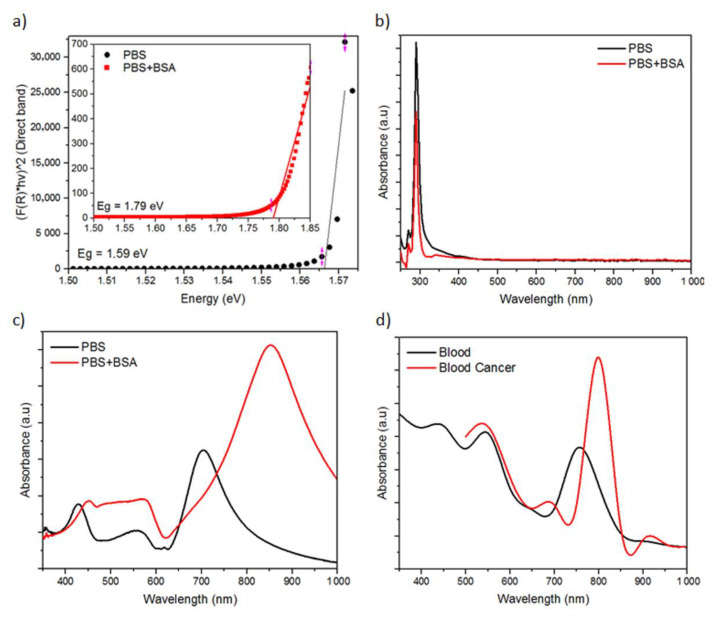
(**a**) The Kubelka–Munk Graph of absorbance measurements of the Ag nanosquare array; (**b**) average value of experimental results of the absorbance of the PBS and PBS+BSA media without the Ag nanosquare array electrode; (**c**) average value of experimental results of the absorbance of the PBS and PBS+BSA media with the Ag nanosquare array electrode; and (**d**) simulation results of the absorbance of the Ag nanosquare array.

The energy gap value was calculated to determine the optical characteristics of the Ag nanosquare array electrode. The energy gap value of the Ag nanosquare array with PBS and PBS+BSA media was identified using the Kubelka–Munk graph. The Kubelka–Munk graph obtained from the results of the experiment is shown in Figure 5a. The smallest energy gap is shown, separating the top part of the valence band and the bottom part of the conduction band [40]. The energy gap value of the Ag nanosquare array with the PBS medium was 1.59 eV, while the energy gap value with the PBS+BSA medium was 1.79 eV. From the results, it can be concluded that the energy gap value of the Ag nanosquare array with the PBS medium is lower than that of the Ag nanosquare array with the PBS+BSA medium. The presence of BSA in the electrode medium can cause bonds between S atoms and Ag electrodes. The energy gap value shows a value that is inversely proportional to the ability of electron transport between the electrode and the electrolyte [41].

Figure 5b shows a comparison of the absorbances between PBS and PBS+BSA media without the use of electrodes from the experiment results. A different peak absorbance occurs in the two media. The peak absorbance occurs at a wavelength of 290.55 nm in the PBS medium and 289.80 nm in the PBS+BSA medium. The existence of a peak at this wavelength is due to the presence of aromatic amino acids in BSA causing maximum absorption in the UV region. The experiment results also show that a higher concentration of BSA results in a higher absorbance value. This higher absorbance value is due to the denser constituent molecules of the solution, which cause more energy from the electromagnetic waves to be absorbed by the constituent molecules of the solution.

The absorbance measurement was also carried out on Ag nanosquare arrays using PBS and BSA as serum albumin by means of dripping and drying the media on the Ag nanostructures. The absorbance measurement of the Ag nanosquare array using the PBS and PBS+BSA medium was carried out several times in which the measurement results were identical and are shown in Figure 5c. The absorbance of the Ag nanosquare arrays was influenced by the Ag electrode, the medium, and the bond between the two, enabling Ag nanosquare arrays to have the ability to absorb EM waves energy in the vis to NIR region. In the experiment using Ag nanosquare arrays, a peak absorbance occurred at a wavelength of 700 nm in the PBS medium and a wavelength of 850 nm in the PBS+BSA medium. Compared to the absorbance measurement without the use of electrodes, the difference in peak absorbance was greater in the absorbance measurement with the use of electrodes. The shift in peak absorbance to different wavelengths occurs due to the different structures of the samples. When the surface of an Ag nanosquare array is coated with albumin, a shift in peak absorbance to a higher wavelength occurs.

The shift of peak absorbance to a larger wavelength due to the addition of BSA to the medium is caused by the interaction between the incoming EM wave and the conduction band of the electrode. This interaction produces a coherence resonance of Ag surface conduction electrons. The binding of BSA to the Ag surface results in a change in the dielectric constant of the surrounding medium which in turn causes a change in the optical characteristics of the EM wave on the Ag surface that is proportional to the change in the concentration of BSA [33,42]. A study conducted by Nonoyama showed a peak optical density at a wavelength of 550 nm for whole blood cells, while a peak optical density for blood cancer was at a wavelength of 800 nm. There is a shift in the wavelength of the peak absorbance to a higher value when compared to whole blood cells [43]. Therefore, serum albumin measurements using the spectrophotometric method are more effective with the use of Ag nanosquare array electrodes compared to measurements without the use of Ag nanosquare array electrodes.

The absorbance measurement was carried out on the Ag nanosquare array to validate the results of the experiment and simulation. The results of the absorbance simulation of the Ag nanosquare array are shown in Figure 5d. The peak absorbance wavelength with the normal blood medium is smaller than the peak absorbance wavelength with the lymphocyte medium. Peak absorbance of the simulation results with the normal blood medium and lymphocyte medium occurred at wavelengths of 774 nm and 800 nm, respectively. Figure 5c,d, which present the results of the experiment and the simulation with different media, show the same pattern in which an increase in the intensity and wavelength of the peak absorbance occurred with respect to an increase in the concentration of serum albumin and the presence of high-concentration white blood cells in the medium as prognostic factors of leukaemia [44,45]. Thus, an optical analysis by means of spectroscopy can be used to monitor the prognosis of blood cancer with the use Ag nanosquare array electrodes.

### 3.3. Electrochemical Analysis of Electrode

CV measurement can be used to detect the electrical response of an arranged Ag nanosquare array and Ag thin film in a medium. After surface activation, Ag nanosquare arrays and thin films are able to experience reduction and oxidation reactions based on the following equations:

Ag + 2OH^−^ → Ag_2_O + H_2_O + 2e^−^ (Oxidation) (1)

**Figure 6:**
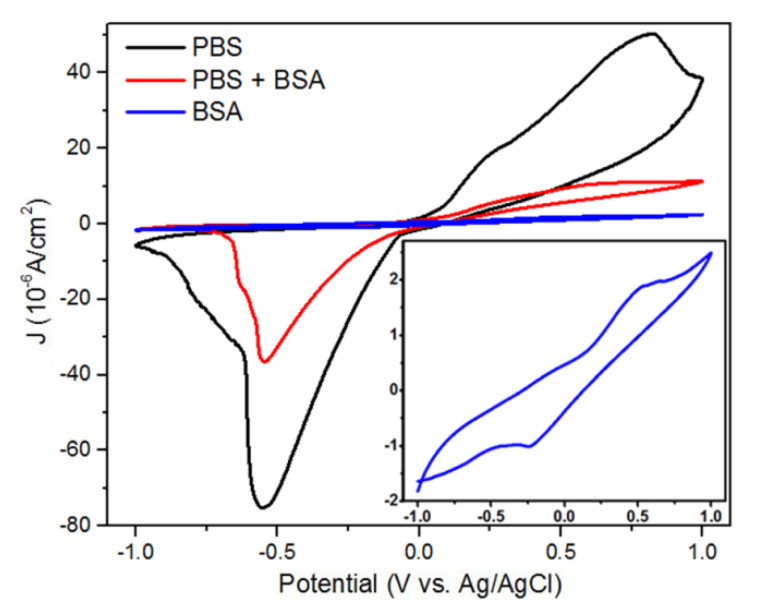
The experiment results of the CV measurement of the Ag thin film using the PBS, PBS+BSA, and BSA media.

Ag_2_O + H_2_O + 2e^−^ → Ag + 2OH^−^ (Reduction) (2)

with peak oxidation and reduction occurring at a voltage of 0.55 V dan −0.55 V, respectively [46,47]. The results of the CV measurement of the PBS, BSA, and PBS+BSA media are shown in Figure 6. At an equivalent concentration of 0.2 mM, the peaks of the oxidation current of the PBS, PBS+BSA, and BSA media were 49.79 μA, 10.21 μA, and 1.90 μA, respectively. The results of the CV measurement show that the peaks of the anodic and cathodic currents decrease as the concentration of BSA in the PBS medium is increased. The presence of BSA may hinder the oxidation reaction of the solution due to BSA decreasing the balance of the solution. Furthermore, BSA also has blocking effect on the surface of electrodes and is able to experience adsorption on the surface of metal electrodes [20]. Therefore, the presence of higher serum albumin within the blood may shift the peak of the oxidation and reduction current to a smaller value.

**Figure 7:**
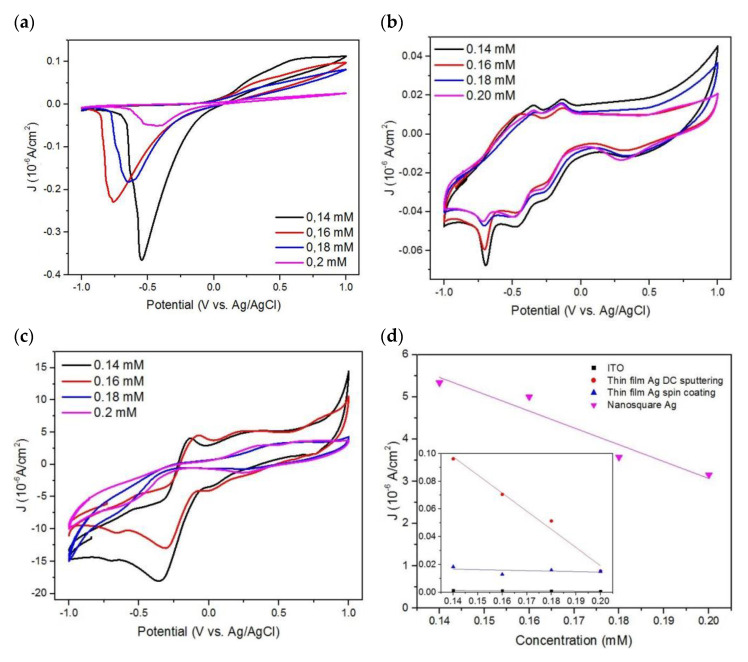
The experiment results of the CV measurement using various concentrations of PBS+BSA as a function of BSA concentration on (**a**) Ag thin-film electrode deposited using DC sputtering, (**b**) Ag thin-film electrode deposited using spin coating, and (**c**) Ag nanosquare array. (**d**) Response of the current towards the change in concentration of the medium.

The correlations between the peaks of oxidation currents and various concentrations of PBS+BSA on Ag thin films deposited using DC sputtering and spin coating and also on the Ag nanosquare array are shown in Figure 7a–c. The figures show a decrease in the peak of the anodic and cathodic currents that is caused by the increase in the PBS+BSA concentration. BSA is a non-electroactive polymer. Therefore, an increase in the concentration of BSA in the medium causes a thicker adsorption layer on the electrode, enabling it to block and inhibit ion transfer which in turn causes a decrease in the peaks of the anodic and cathodic currents.

The response of the current towards the change in concentration of the medium is shown in Figure 7d. Due to the change in the peak of the anodic and cathodic currents that is in line with the change in the concentration of the medium, the response towards the change in the peaks of the currents can be used to identify the sensitivity of the electrode in detecting the concentration of the medium. The sensitivity of the electrode can be determined from the change in the peak of the current towards the change in concentration [48]. From Figure 7, it is proven that the addition or deposition of Ag thin films or nanosquare arrays on top of the ITO glass may increase the peaks of the anodic and cathodic currents and also increase the sensitivity of the electrode in detecting the concentration of the protein. This is caused by the conductivity of the ITO glass being much smaller to that of Ag, and as a result, Ag thin films and nanosquare arrays can distribute electrons easier in the reduction and oxidation process [49]. A calculation of the coefficient of variation on various concentrations of BSA was also carried out and is shown in Figure S2. It can be seen from Figure S2 that, in general, the greater the concentration of BSA, the greater the coefficient of variation. This shows that the greater the concentration of BSA, the greater the risk of uncertainty from the measurement due to various protein–electrode interactions.

The sensitivities of the Ag thin-film electrode deposited using DC sputtering and the Ag thin film deposited using spin coating were 0.022 µA mM^−1^cm^−2^ and 1.308 µA mM^−1^cm^−2^ in a concentration of 0.12 mM up to 0.2 mM, while the sensitivity of the Ag nanosquare array electrode was 39.917 µA mM^−1^cm^−2^ in a concentration of 0.12 up to 0.2 mM. Ag thin films deposited using spin coating possess a lower sensitivity than that of thin films deposited using DC sputtering. This is caused by the inhomogeneous distribution of Ag in the thin films, thus increasing the resistance and decreasing the ability of the surface of the electrode to bind BSA. The performance of the Ag nanosquare array was better than Ag thin films based on the sensitivity value that is dependent on the area of the electrode. A larger area of the nanosquare array provides a larger surface for diffusion between electrolytes and electrodes, which causes the occurrence of reduction and oxidation reactions.

**Figure 8:**
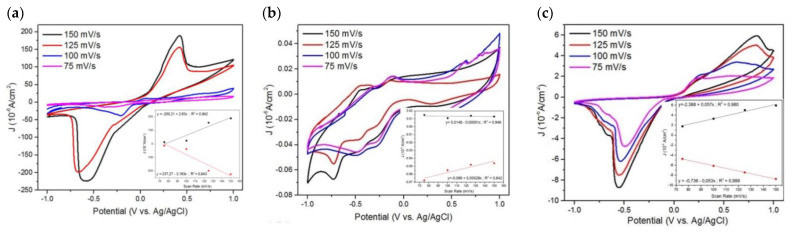
The experiment results of the CV measurement and peaks of oxidation and reduction currents as a function of scan rate on (**a**) the Ag thin-film electrode deposited using DC sputtering, (**b**) the Ag thin-film electrode deposited using spin coating, and (**c**) the Ag nanosquare array electrode.

The measurement result on the effect of scan rate towards the peaks of anodic and cathodic currents is in accordance to the Rendles–Sevcik equation where the peak values of anodic and cathodic currents are proportional to the scan rate. In the reduction and oxidation process of CV, currents that passed through the electrode was limited by the diffusion of atoms on the surface of the electrode. The number of atoms that were diffused was influenced by the concentration gradient of the solution near the electrode. The concentration gradient value was influenced by the concentration of the solution near the electrode and the rate of diffusion of the electrolytes through the solution [50]. Therefore, a faster change in voltage causes a larger concentration gradient near the electrode and produces a larger current. The inset plots of Figure 8a–c shows the response to the change in the peaks of anodic and cathodic currents throughout the redox process of the solution. It can be seen in Figure 8a–c that there is a correlation in the change of peak currents between the reduction and oxidation process. This shows that there is a good diffusion-controlled process at the electrode when the reaction takes place. The total reaction rate depends on the ion diffusion process from the solution to the surface of the electrode [48].

The results of the kinetic performance study of the Ag thin film deposited using DC sputtering, the Ag thin film deposited using spin coating, and the Ag nanosquare array in detecting the concentration of serum albumin within the solution using varying scan rates are shown in Figure 8a–c, respectively. The suitability value between changes in the peaks of oxidation and reduction currents in the Ag thin film deposited using DC sputtering was greater than the suitability value in the Ag thin film deposited using spin coating. This is due to the better diffusion control in the Ag thin film deposited using DC sputtering as a result of its more homogeneous structure. It can be seen that the results of the CV measurement of the Ag nanosquare array electrode exhibits the same pattern to that of Ag thin films deposited by DC sputtering and spin coating. Thus, it can be concluded that a larger scan rate results in bigger peaks of the anodic and cathodic currents. The shift in the peaks of the anodic and cathodic currents and the change in value of scan rates between the reduction and oxidation process of the Ag nanosquare array are more similar than those of Ag thin films. This proves that the Ag nanosquare array possess a better diffusion control. A better diffusion control in the Ag nanosquare array is caused by a larger surface of the Ag nanosquare array where the diffusion process occurs; thus, the diffusion process takes place more smoothly. The calculation of the coefficient of variation in Figure S3 shows that an increase in the coefficient of variation occurs with respect to an increase in scan rate. This is due to the better diffusion process between the electrode and BSA at lower scan rates.

**Figure 9:**
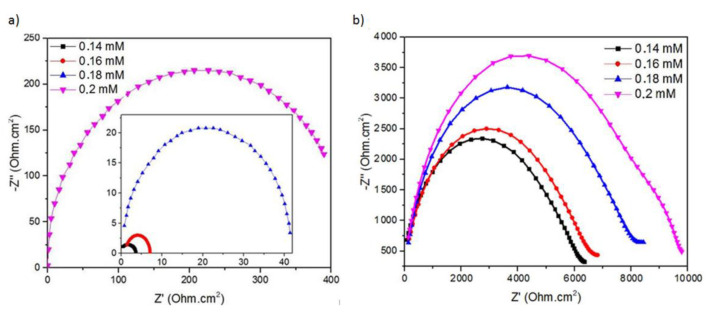
The experiment results of the EIS measurement with varying concentrations of the PBS+BSA medium using (**a**) the Ag thin film and (**b**) the Ag nanosquare array.

The measurement of the Ag thin film and nanosquare array electrodes as a prognosis monitoring sensor for blood cancer based on the concentration of serum albumin was also carried out by analysing the results of the EIS measurements. The results of the EIS measurements on Ag thin films and Ag nanosquare arrays using various concentrations of BSA are shown in Figure 9a,b, respectively. The system that was comprised of ITO glass substrate, Ag thin films or Ag nanosquare arrays, and PBS+BSA solution was equivalent to the sequence of R_s_ as the solution resistance, C_p_ as the model equal to ITO/Ag double layer capacitance, and R_ct_ as the ITO/Ag charge transfer resistance [51]. R_ct_ can be used to evaluate the bond between electrolytes and biosensors because it is based on the Faraday impedance that is measured with the presence of a redox mediator [52]. The increase in the R_ct_ value can be shown by the increase in the diameter of the half-circle of the Nyquist graph. The increase in the diameter of the Nyquist graph is in line with the increase in the capacitance and resistance of the surface of the sensor. The binding of electrolyte to the surface of the biosensor may cause an increase in the impedance of the electrode [53]. The increase in the R_ct_ value, identified from the Nyquist graph, which is in line with the increase in the concentration of BSA, indicating an increase in BSA adsorption at the electrode [14]. A slight increase in the concentration of BSA in the solution can significantly increase the R_ct_ value. This occurs due to the addition of the BSA molecule that contains cysteine which has an -SH chain. Thus, an increase in the concentration of BSA will significantly increase the number of Ag-S bonds, which reduces the electron transport ability between BSA and the electrode [41].

Based on the comparison of the R_ct_ values between the Ag thin film and the Ag nanosquare array, it can be identified that a change in the R_ct_ value with respect to a change in the concentration of the solution is smaller on the Ag thin film than on the Ag nanosquare array. The change in R_ct_ value with respect to the change in the concentration of the solution on the Ag thin film and the Ag nanosquare array were 6593.76 Ohm·cm^2^/mM and 69,000 Ohm·cm^2^/mM, respectively. A larger change in the R_ct_ value with respect to the concentration of the electrolyte shows that the electrode becomes more sensitive in detecting the electrolyte concentration using the EIS method. In this study, it has been proven that the Ag nanosquare array is more sensitive than the Ag thin film. This is caused by the larger surface of the Ag nanosquare array electrode compared to the Ag thin film, in which the surface is used to bond with electrolyte. Therefore, the bonding of electrode with electrolyte on the larger surface of Ag nanosquare arrays when a change in the concentration of the solution occurs has a greater influence in changing the R_ct_ value.

The electrochemical method has better sensitivity compared to the spectroscopic method due to the change in the signal from the measurement with respect to the change in concentration of the protein biomarker. The electrochemical method is better because the electrode can bind to proteins and detect specific molecules that make up the solution [14,48]. The spectroscopic method can detect changes in the concentration of solutions but is not specific for certain types of solutions due to the possibility of the overlapping of absorbance signals between biological molecules and other molecules [7]. Spectroscopic testing possesses a faster measurement speed than electrochemical testing. The spectroscopic method is more accurate in detecting biological material on biological media that are easily damaged compared to the electrochemical method.

## 4. Conclusions

The fabrication of Ag thin film and Ag nanosquare array electrodes were successfully carried out using the DC sputtering and spin coating deposition method. The electrical and optical characteristics of Ag thin films and the Ag nanosquare arrays were systematically investigated using varying concentrations of albumin in its medium. The results of the experiment and simulation showed a shift of peak absorbance towards a bigger wavelength as much as 26 to 150 nm at the Ag nanosquare array electrode with respect to an increase in the concentration of prognostic factors, namely BSA and leukocytes. The electrochemical test indicated an increase in the peaks of anodic and cathodic currents with respect to a decrease in the concentration of the PBS+BSA solution in the CV measurement. The Ag thin layer deposited by spin coating has a lower sensitivity in testing the concentration of BSA by the electrochemical method compared to the Ag thin layer deposited by DC sputtering. In addition, the Ag nanosquare array electrode was more sensitive when detecting the difference in concentration of BSA using both the spectroscopic and electrochemical methods compared to the Ag thin-film electrode deposited using DC sputtering and the Ag thin film deposited using spin coating. The Ag nanosquare array is a better electrode to be used in the prognosis monitoring of blood cancer.

## Data Availability

Not applicable.

## Supplementary Materials

The Supplementary Materials are available online at https://www.mdpi.com/article/10.3390/nano11113108/s1. Figure S1: Simulation results of absorbance (a) thin film and nanosquare (b) 300 nm, (c) 250 nm, (d) 200 nm, (e) 150 nm, and (f) 100 nm on normal blood medium and cancer blood. Figure S2: The coefficient of variation as measurement result of the cyclic voltammetry using varying concentration. Figure S3: The coefficient of variation as measurement result of the cyclic voltammetry using varying scan rate.
