# Supplementary material for: A Comparison between Silver Nanosquare Arrays and Silver Thin-Films as a Blood Cancer Prognosis Monitoring Electrode Design Using Optical and Electrochemical Characterization
Source: Nanomaterials (Basel). 2021 Nov 18;11(11):3108. doi: 10.3390/nano11113108 (PMC8625830; doi:10.3390/nano11113108)
Supplement: Supplementary file 1 [file nanomaterials-11-03108-s001.zip › nanomaterials-1449364-supplementary.pdf]

*Supplementary Materials (SM)*

# A Comparison between Silver Nanosquare Arrays and Silver Thin-Films as a Blood Cancer Prognosis Monitoring Electrode Design Using Optical and Electrochemical Characterization


**Nasori Nasori [1,2,*], Ulya Farahdina [1], Vinda Zakiyatuz Zulfa [1], Miftakhul Firdhaus [1], Ihwanul Aziz [3], Darsono Darsono [3], Dawei Cao [4], Zhijie Wang [5], Endarko Endarko [1] and Agus Rubiyanto [1]**

1 Laboratory Medical Physics and Biophysics, Department of Physics, Faculty of Sciences and Data Analytic, Institut Teknologi Sepuluh Nopember, Surabaya 60111, Indonesia; ulyafarahdina06@gmail.com (U.F.); vzakiyatuz@gmail.com (V.Z.Z.); mfirdauz8@gmail.com (M.F.); endarko@physics.its.ac.id (E.E.); arubi@physics.its.ac.id (A.R.)
2 Occupational and Safety Department, Nahdlatul Ulama University of Surabaya, Surabaya 60237, Indonesia
3 Center for Accelerator Sciences and Technology, Yogykarta 60101, Indonesia; ihwanul@batan.go.id (I.A.); b_darsono@batan.go.id (D.D.)
4 Department of Physics, Faculty of Sciences, University of Jiangsu, Zhenjiang 212013, China; dwcao@ujs.edu.cn
5 Semiconductor Materials Science Key Laboratory, Semiconductors Institute, Chinese Sciences Academy, Beijing 100083, China; wangzj@semi.ac.cn
* Correspondence: nat.nasori@physics.its.ac.id


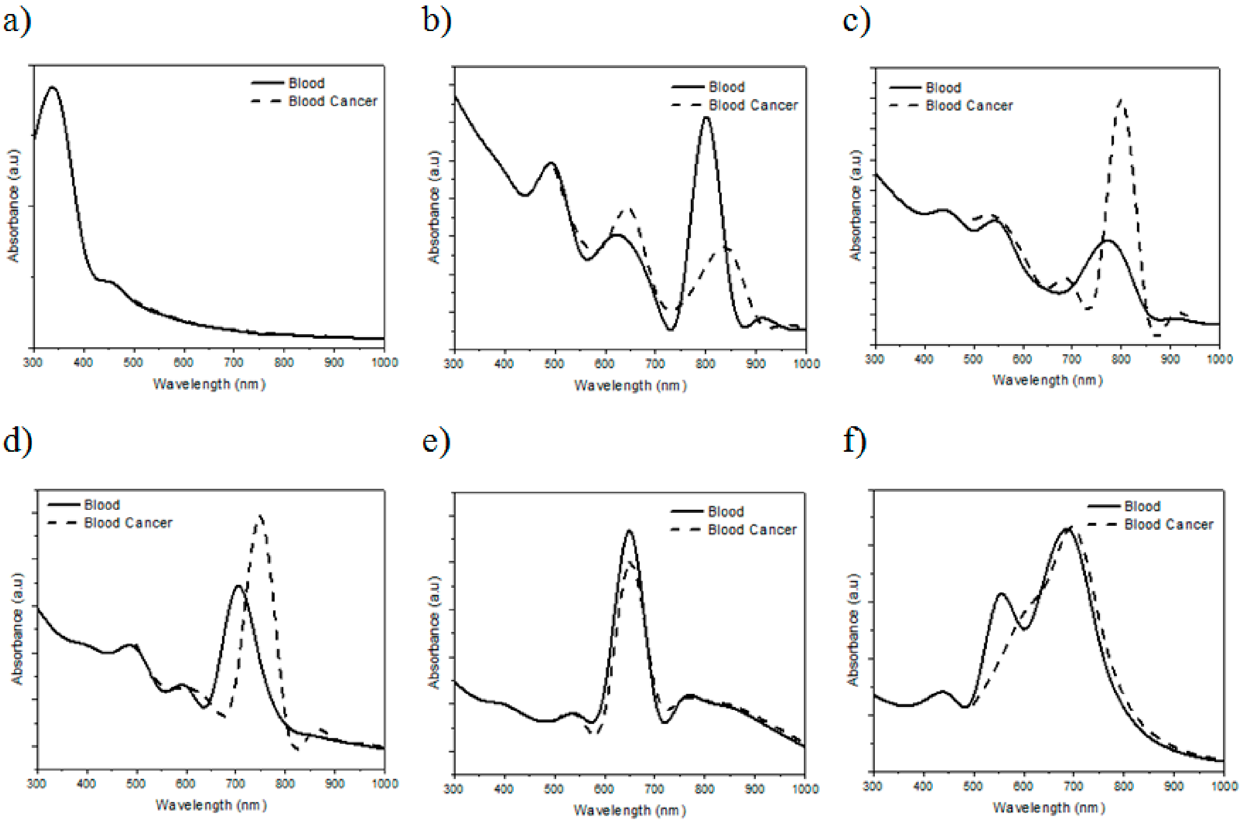


**Figure S1.** Simulation results of absorbance (a) thin layer and Nanosquare (b) 300 nm, (c) 250 nm, (d) 200 nm, (e) 150 nm, and (f) 100 nm on Normal Blood Medium and Cancer Blood

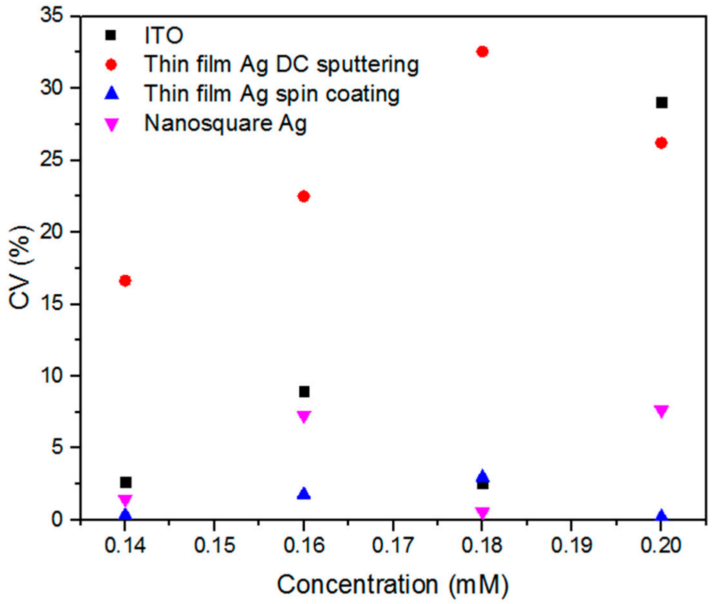


**Figure S2.** Coefficient of Variation as Measurement Result of The Cyclic Voltammetry Using Varying Concentration.

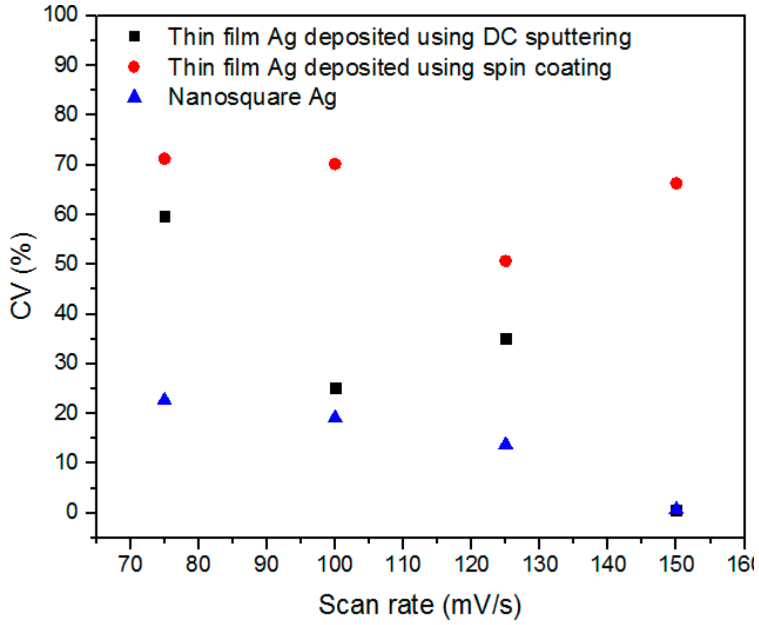


**Figure S3.** Coefficient of Variation as Measurement Result ff The Cyclic Voltammetry Using Varying Scan Rate.
